# Spherical Activated Carbons with High Mechanical Strength Directly Prepared from Selected Spherical Seeds

**DOI:** 10.3390/ma11050770

**Published:** 2018-05-10

**Authors:** Ana Amorós-Pérez, Laura Cano-Casanova, Mohammed Ouzzine, Mónica Rufete-Beneite, Aroldo José Romero-Anaya, María Ángeles Lillo-Ródenas, Ángel Linares-Solano

**Affiliations:** MCMA Group, Department of Inorganic Chemistry and Materials Institute, University of Alicante, E-03080 Alicante, Spain; ana.amoros@ua.es (A.A.-P.); laura.cano@ua.es (L.C.-C.); ouzzine_mohamed@yahoo.fr (M.O.); monica.rufete@ua.es (M.R.-B.); ajromero@ua.es (A.J.R.-A.); linares@ua.es (A.L.-S.)

**Keywords:** spherical seeds, spherical activated carbons, activation, microporosity, mechanical properties

## Abstract

In the present manuscript, the preparation of spherical activated carbons (SACs) with suitable adsorption properties and high mechanical strength is reported, taking advantage of the retention of the spherical shape by the raw precursors. An easy procedure (carbonization followed by CO_2_ activation) has been applied over a selection of three natural seeds, with a well-defined spherical shape and thermal stability: *Rhamnus alaternus* (RA), *Osyris lanceolate* (OL), and *Canna indica* (CI). After the carbonization-activation procedures, RA and CI, maintained their original spherical shapes and integrity, although a reduction in diameter around 48% and 25%, respectively, was observed. The porosity of the resulting SACs could be tuned as function of the activation temperature and time, leading to a spherical activated carbon with surface area up to 1600 m^2^/g and mechanical strength similar to those of commercial activated carbons.

## 1. Introduction

Spherical activated carbons (SACs) are very interesting materials, which are attracting great attention because of their outstanding physical properties, such as wear resistance, mechanical strength, good adsorption performance, purity, low ash content, smooth surface, good fluidity, good packaging, low pressure drop, high bulk density, high micropore volume and tunable pore size distribution [1,2,3,4,5]. All these features make SACs suitable for various applications like blood purification, catalysts support, chemical protective clothing [2,6,7], in adsorption processes; both in gas phase (e.g., toluene, CO_2_, CH_4_ and H_2_) [5,8,9,10] and solution (e.g., phenol) [11], as supercapacitors [12,13], in medicine for poison adsorption in living organisms [14], as catalyst supports for hydrogenation reactions [15,16], etc.

SACs can be prepared using several methods: by polymerization reactions [17], by agglomeration from mixtures of resin and activated carbon [18] or by hydrothermal synthesis [19,20,21,22,23]. All these methods imply the use of expensive or synthetic precursors (such as aerogels [17], divinylbenzene-derived polymers [24] and urea/formaldehyde resin [25]). However, nowadays it is common to look for cheaper precursors, such as coals [4], lignocellulosic materials [19,20,21] and carbohydrates [22,26,27,28,29].

**Figure 1:**
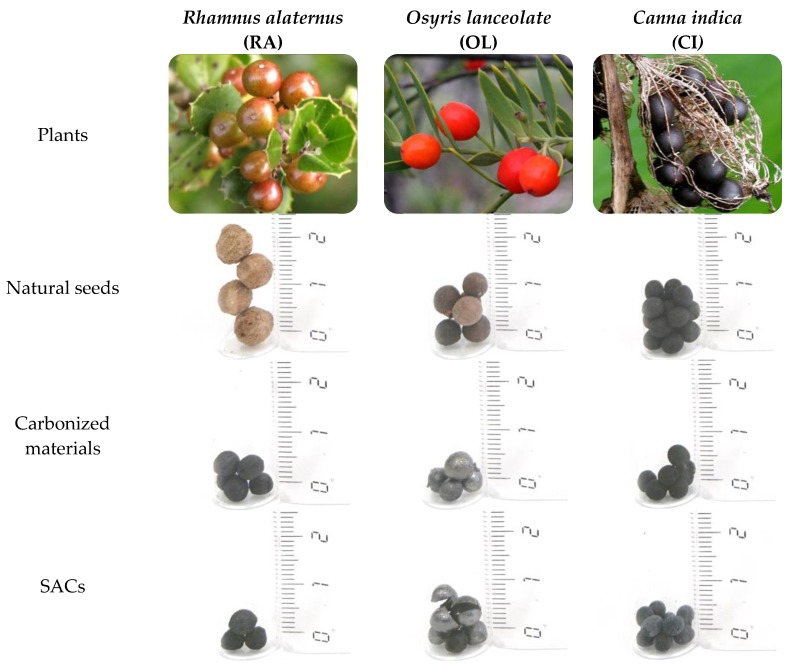
Natural seeds used as precursors for SACs preparation, together with the carbonized and activated spherical materials prepared from them.

Herein we present the preparation of SACs with high mechanical strength and tunable porosity from spherical seeds using an easy, cheap and a well-known method. This simple route allows the valorization of inexpensive and available biomass precursors, such as not edible seeds, to convert them in potentially useful and valuable materials like SACs. In particular, we focused our interest on the selected seeds that combine simultaneous spherical shape and thermal stability, and cover a wide range of diameters (from 1 to 7 mm). It should be highlighted that the size of the final materials would depend on the size of the used precursor. Among the tested spherical seeds, which accomplish these requirements (Table 1), the study was focused on three of them: *Rhamnus alaternus* (RA), *Osyris lanceolate* (OL), and *Canna indica* (CI) (Figure 1).

## 2. Materials and Methods

### 2.1. Methodology

#### 2.1.1. Carbonization Process

All the seeds were initially dried in an oven at 110 °C for 3 h. For the carbonization process, 2 g of such dried seeds were heated up to 850 °C, held for 2 h, in a horizontal furnace with N_2_ flow of 300 mL/min, and a heating rate of 5 °C/min. The corresponding carbonization yields are summarized in Table 2.

#### 2.1.2. Activation Process

Carbonized seeds were activated using CO_2_ in order to develop their porosity, using a CO_2_ flow of 80 mL/min. To study the effect of temperature and time on the activation process, the samples were heated at 5 °C/min up to different temperatures: 800, 850, or 880 °C, and such temperatures were maintained for various fixed times, as described in Table 3.

### 2.2. Characterization

#### 2.2.1. Morphology

Morphology of the original, carbonized and activated samples was characterized by Scanning Electron Microscopy (SEM) in a JSM-840 microscope (JEOL, Tokyo, Japan) with a scintillator–photomultiplier type secondary electron detector.

#### 2.2.2. Surface Area and Pore Volumes

Textural characterization of precursors, carbonized and activated materials was performed using N_2_ adsorption at −196 °C [31] and CO_2_ at 0 °C [32] in a volumetric Autosorb-6B apparatus from Quantachrome. Before analysis, the samples were degassed at 250 °C for 4 h. The BET equation was applied to the nitrogen adsorption isotherm in the low-pressure region (relative pressure between 0.05–0.25) to get the apparent BET surface area, S_BET_ [30]. The Dubinin–Radushkevich equation was applied to the nitrogen adsorption isotherm to determine the total micropore volume (V_DR_ (N_2_) corresponding to micropores of size below 2 nm) and to the carbon dioxide adsorption isotherms to determine narrow micropore volume (V_DR_ (CO_2_), corresponding to micropores of size below 0.7 nm) [33]. Mesopore volume, V_meso_, corresponding to pores between 2 and 20 nm, was estimated from N_2_ adsorbed as liquid at P/P_0_ = 0.9 minus the volume adsorbed at P/P_0_ = 0.2 [30]. The difference between V_DR_ (N_2_) and V_DR_ (CO_2_) was calculated as an estimation of the micropore size distribution [30,31].

#### 2.2.3. Mechanical Properties

**Figure 2:**
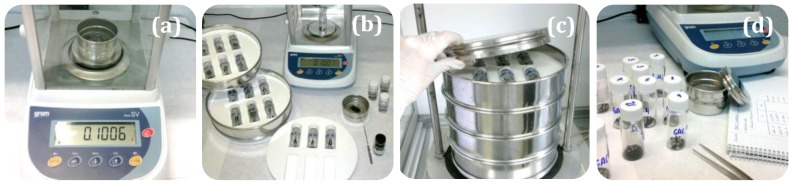
Depiction of materials and procedure for the mechanical strength evaluation of the samples: (**a**) a weighted sample was sieved using in 300 μm sieve and put in a vial; (**b**) 15 steel balls were also incorporated in the vial, which was then placed in a polymer mold; (**c**) the molds were placed in the sieve shaker during 20 min; (**d**) the sample was sieved again in a 300 μm sieve and the residue (the sample not converted to dust in the sieving step) was collected and weighed to calculate the sample remaining mass percentage (SRM%).

The mechanical strength, defined as SRM%, was estimated by a method developed in our laboratory that consists of the evaluation of the sample mass remaining in a sieve after vigorous shaking (Figure 2). Thus, for each test, a known quantity of material was put in a cylindrical vial together with 15 stainless steel balls (Figure 2a). These vials were placed horizontally in a polymer mold used as immobilizing support (Figure 2b), which was placed in an electromagnetic sieve shaker CIPSA RP08 Ø200/203 for 20 min at power number 8 (equivalent to the shaking speed of 1.8 mm of vibration amplitude per second) (Figure 2c). Then, the samples were sieved using a sieve (300 μm) and the resulting residue (the sample not converted to dust in the sieving step) was weighed (Figure 2d). The mechanical strength was expressed as the percentage of the remaining mass after sieving (SRM%). The validation of this method was performed by analyzing the mechanical properties of several commercial activated carbons (Table 4), and such values were used as reference to confirm that the mechanical properties of our SACs are similar to those of their commercial counterparts. Note that the SRM values for the selected commercial ACs are in the range between 72% and 97%. The analysis of the mechanical properties was performed for the precursors, carbonized materials (Table 2) and for the activated ones (Table 3).

## 3. Results

**Figure 3:**
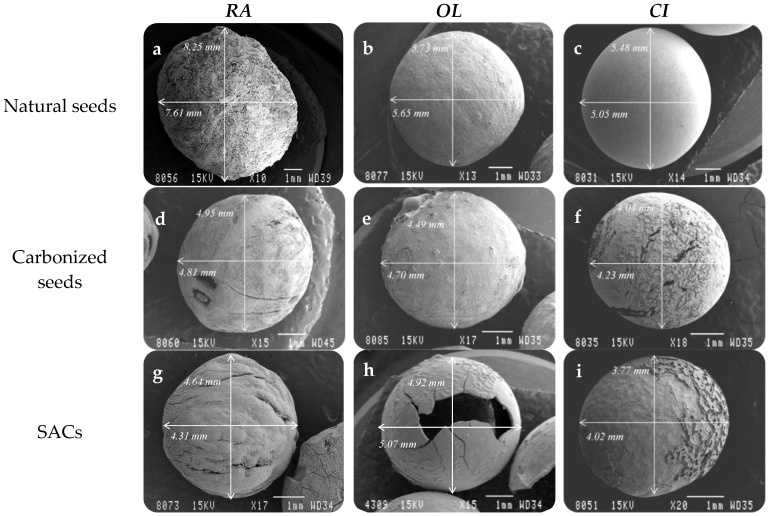
SEM images of precursors, carbonized materials and final SACs. Experimental preparation conditions of the materials: (**a**–**c**) dried at 110 °C for 3 h; (**d**–**f**) carbonized at 850 °C for 2 h in 300 mL/min N_2_ flow; (**g**) activated at 800 °C for 30 h using 80 mL/min CO_2_ flow; (**h**) activated at 800 °C for 2 h in 80 mL/min CO_2_ flow; (**i**) activated at 800 °C for 5 h in 80 mL/min CO_2_ flow.

Figure 3 shows that the retention of the desired original spherical shape during the carbonization process was achieved for these three seeds, though this step generally led to a decrease in the diameter of the materials. Such variation depends on the type of seed: for RA the size was significantly reduced (around 3 mm, which represents 40% reduction), while CI and OL were only slightly shortened (in both cases about 1 mm, around 20%). This could be related with the intrinsic natural differences in the composition of the seeds.

Table 2 reports carbonization yields and values of mechanical properties (SRM%) for CI, RA, and OL carbonized seeds, together with SRM values for the precursors, as reference. It shows that: (i) the carbonization yields ranged from 21 to 30%, which is in the range of typical values expected for lignocellulosic materials [34]; (ii) micropore volumes determined by CO_2_ adsorption were larger than those measured by N_2_, indicating that the mean micropore sizes were below 0.7 nm [31], and (iii) SRM values were higher than 98.7%, indicating that the samples possess high mechanical resistance. Such porous texture, together with the mechanical properties, made these carbonized materials potentially useful as spherical carbon molecular sieves.

The conditions of the activation process were also optimized in order to obtain similar burn-off percentage (around 30%) and maintain the spherical morphology. Figure 3 shows that RA and CI seeds retained their original shape after activation, and their sizes were minimally affected (around 0.5 mm (8% reduction with respect to the size) before the activation, and 0.2 mm (5%), respectively). Only OL seeds were broken after activation, and this occurred for all the explored activation conditions. Hence, from OL only spherical carbonized materials could be prepared. It is important to mention that the carbonized and activated materials from both RA and CI remained physically intact (without cracks) and the same occurred for carbonized OL, whereas only the material obtained from OL, after the activation process showed cracks, that can be visually distinguished.

With respect to the activation yields (Table 3), CI was the more reactive candidate, since a shorter activation time was required to get 33% burn-off. For the RA precursor, as expected, the burn-off percentage at constant temperature increases proportionally with the reaction time. Interestingly, the desired activation percentage (33%) could also be achieved for RA using higher temperatures (850 °C instead of 800 °C) and shorter times (10 h instead of 40 h).

Regarding the textural properties, Table 3 shows that, for RA seeds, although there exists a linear relationship between the activation time and the burn-off percentage, no direct correlation was found when analyzing the effect of the activation time on the porosity development. For this precursor, the same burn-off percentage, 33%, and similar surface area values (about 880 m^2^/g) have been obtained using different combinations of activation temperature and time. Low activation times (10 and 30 h) led to materials with the mean micropore sizes below 0.7 nm, whereas the micropore size was around that value for larger activation time and temperature.

Similar experimental conditions screening for CI precursor highlighted that higher adsorption capacities can be developed from it, which reached 1616 m^2^/g when treating up to 880 °C for 3 h. For the CI activated material with surface area above 1600 m^2^/g, the fact that total micropore volume determined by N_2_ adsorption is much larger than that measured by CO_2_, is indicative of the average pore size above 0.7–1 nm [30].

**Figure 4:**
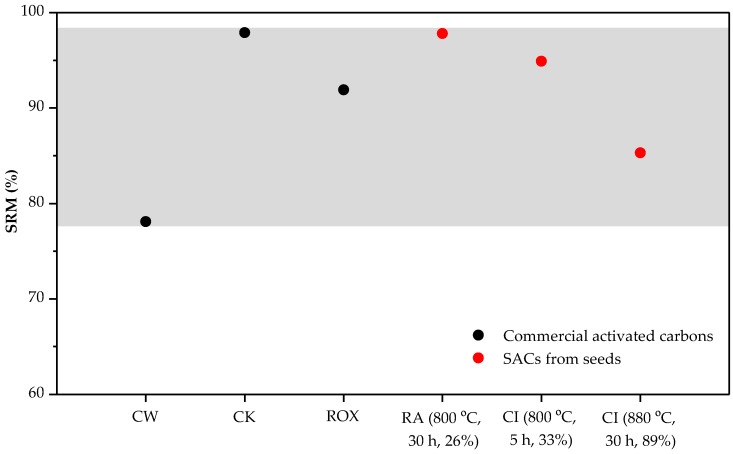
SRM values for commercial activated carbons (black color) and for some activated seeds prepared in this work (orange color).

By comparing SRM values for natural and carbonized materials in Table 2, it can be observed that natural precursors show slightly higher mechanical strength than the corresponding carbonized spheres. Table 3 contains the SRM values for SACs, indicating that their mechanical properties are only slightly reduced after the activation process. This is probably due to the high number of heteroatoms linked to the carbon material, and eliminated during the activation [35]. However, SRM values are in the range of 95% for samples with areas around 800 m^2^/g, and about 85% when the BET surface area surpasses 1600 m^2^/g, which indicated that the samples generally display significant mechanical properties that lie in the range of the selected common commercial references (CW, CK and ROX) (Table 4 and Figure 4).

## 4. Conclusions

In this work spherical activated carbons with high mechanical strength and well-developed porosity were prepared while maintaining the spherical shape of the natural seeds, selected as carbon precursors. The three reported candidates: RA, OL and CI, could be successfully converted into spherical activated carbon materials using a well-known, simple and cheap method and, additionally, CI and RA maintained their original spherical shapes and integrity all along the activation process, avoiding breakage. Their diameter sizes were notably reduced after the carbonization step and only slightly affected by the activation process. The mechanical properties for all the activated materials were found to be similar to those of common commercial activated carbons with different morphologies (granular, spherical and pellets). Interestingly, depending on the precursor and/or on the activation conditions, significant differences in porosity development and micropore size distributions were obtained, reaching specific surface areas up to 1600 m^2^/g. The interesting properties of the prepared materials, together with their spherical morphology, make them interesting candidates for many applications.

## Figures and Tables

**Table 1:** Common and scientific names of the selected spherical seeds and their diameters.

Common Name	Scientific Name	Mean Diameter (mm)
Poppy	*Papaver rhoeas*	1
Amaranth	*Amaranthus hypochondriacus*	1
Millet	*Panicum miliaceum*	2
Mustard	*Sinapis alba*	3
Black pepper	*Piper nigrum*	4
False pepper	*Schinus molle*	4
Palm	*Phoenix dactylifera*	5
Indian shot	*Canna indica*	5
African sandalwood	*Osyris lanceolate*	5
Phoenicean juniper	*Juniperus phoenicea*	6
Mediterranean buckthorn	*Rhamnus alaternus*	7
Prickly juniper	*Juniperus oxycedrus*	7

**Table 2:** Mechanical strength (expressed as the percentage of the remaining mass after sieving, SRM%, see Section 2.2.3.) of the natural seeds and carbonization yields, textural properties and mechanical strength (SRM%) of the materials after carbonization.

Precursor	SRM ^a^ (%)	Yield ^b^ (%)	V_DR_ (N_2_) ^c^ (cm^3^/g)	V_DR_ (CO_2_) ^d^ (cm^3^/g)	SRM ^e^ (%)
*RA*	99.1	30	0.01	0.18	98.8
*OL*	99.9	21	0.01	0.19	99.4
*CI*	99.0	22	0.02	0.20	98.7

^a^ SRM, remaining mass after sieving the natural spherical seeds, as percentage. ^b^ Yield, yield of carbonization process, as percentage. ^c^ V_DR_ (N_2_), total micropore volume, obtained applying the Dubinin-Raduskevich method to data of N_2_ adsorption isotherm at −196 °C. ^d^ V_DR_ (CO_2_), narrow micropore volume, obtained applying the Dubinin-Raduskevich method to data of CO_2_ adsorption isotherm at 0 °C. ^e^ SRM, remaining mass after sieving the carbonized materials, as percentage.

**Table 3:** Activation conditions, activation percentages, SRM values and textural properties of some activated samples.

Precursor	T (°C)	t (h)	Burn-off (%)	S_BET_^a^ (m^2^/g)	V_DR_ (N_2_) ^b^ (cm^3^/g)	V_DR_ (CO_2_) ^c^ (cm^3^/g)	V_meso_^d^ (cm^3^/g)	SRM ^e^ (%)	V_N2_–V_CO2_^g^ (cm^3^/g)
*RA*	800	10	6	492	0.20	0.25	0.03	NM^f^	< 0
	800	30	26	812	0.28	0.36	0.02	97.8	< 0
	800	40	33	889	0.40	0.33	0.03	NM^f^	0.07
	850	10	33	874	0.39	0.37	0.02	NM^f^	0.02
*CI*	800	5	33	856	0.39	0.35	0.05	94.9	0.04
	880	3	89	1616	0.64	0.37	0.19	85.3	0.27

^a^ S_BET_, BET surface area, obtained applying the BET method to data of N_2_ adsorption isotherm at −196 °C. ^b^ V_DR_ (N_2_), total micropore volume, obtained applying the Dubinin–Raduskevich method to data of N_2_ adsorption isotherm at −196 °C. ^c^ V_DR_ (CO_2_), narrow micropore volume, obtained applying the Dubinin–Raduskevich method to data of CO_2_ adsorption isotherm at 0 °C. ^d^ V_meso_, mesopore volume, obtained from N_2_ adsorbed as liquid at P/Po = 0.9 minus the adsorbed volume at P/P_0_ = 0.2 [30]. ^e^ SRM, remaining mass after sieving the activated materials, as percentage. ^f^ NM: not measured. ^g^ V_N2_–V_CO2_, difference between V_DR_ (N_2_) and V_DR_ (CO_2_).

**Table 4:** Textural properties and SRM values of some commercial activated carbons.

Name	Commercial Name	Morphology and Size	S_BET_^a^ (m^2^/g)	V_DR_ (N_2_) ^b^ (cm^3^/g)	V_DR_ (CO_2_) ^c^ (cm^3^/g)	V_meso_^d^ (cm^3^/g)	SRM (%)
CW	Mead Westvaco, WVA1100	Granular (10 × 25 mesh)	1796	0.72	0.34	0.42	72
CK	Kureha Corporation carbon from petroleum pith	Spherical (0.75 µm)	1185	0.57	0.42	0.02	97
ROX	NORIT^®^ ROX	Pellets (0.8 mm)	1354	0.60	0.40	0.07	92

^a^ S_BET,_ BET surface area, obtained applying the BET method to data of N_2_ adsorption isotherm at −196 °C. ^b^ V_DR_ (N_2_), total micropore volume, obtained applying the Dubinin-Raduskevich method to data of N_2_ adsorption isotherm at −196 °C. ^c^ V_DR_ (CO_2_), narrow micropore volume, obtained applying the Dubinin–Raduskevich method to data of CO_2_ adsorption isotherm at 0 °C. ^d^ V_meso_, mesopore volume, obtained from N_2_ adsorbed as liquid at P/P_0_ = 0.9 minus the adsorbed volume at P/Po = 0.2 [30].
